# Effects of continuous supplementation of *Acanthopanax senticosus* Harms on the cardiac autonomic function of community-dwelling elderly individuals during resting and standing tests: a randomized controlled trial

**DOI:** 10.3389/fcvm.2024.1336676

**Published:** 2024-03-08

**Authors:** Takeru Sato, Takumi Aoki, Yuki Ito, Kan Oishi, Masaki Fujishima, Eri Okumura, Kojiro Ishii

**Affiliations:** ^1^ Graduate School of Health and Sports Science, Doshisha University, Kyo-Tanabe, Japan; ^2^ Faculty of Education, Miyagi Gakuin Women’s University, Sendai, Japan; ^3^ College of Life and Health Sciences, Chubu University, Kasugai, Japan; ^4^ Sun Chlorella Corp., Kyoto, Japan; ^5^ Faculty of Health and Sports Science, Doshisha University, Kyo-Tanabe, Japan

**Keywords:** *Acanthopanax senticosus* Harms, cardiac autonomic function, standing test, elderly individuals, dizziness

## Abstract

**Background:**

Cardiac autonomic function (CAF) decreases with aging, and *Acanthopanax senticosus* Harms (ASH) consumption reportedly induces anti-stress effects. This study aimed to assess the effect of continuous supplementation of ASH on CAF during resting and standing tests in the elderly population.

**Methods:**

This double-blind, randomized controlled trial was conducted in the morning in a laboratory setting and was carried out between June 2017 and July 2017 at Kambaikan, Doshisha University (Karasuma-higashi-iru, Imadegawa-dori, Kamigyo-ku, Kyoto 602-8580, Japan). In total, 28 community-dwelling elderly individuals (mean ± standard deviation = 72.5 ± 4.5 years) were included. Each subject was instructed to consume ASH or placebo supplements twice daily for 4 weeks. An autonomic reflex orthostatic tolerance recorder was used to measure CAF in pre- and post-intervention phases. Parameters were measured in a seated position and included coefficient of variation of R-R intervals (CVRR), low frequency (LF), high frequency (HF), LF/HF ratio, blood pressure, and heart rate (HR). Changes in each parameter were evaluated before and after standing. All parameters were defined as the difference between the mean value obtained in a standing position for 2 min and that obtained in a 2-min seated position.

**Results:**

A two-way analysis of variance revealed a significant group-time interaction effect on CVRR, HF, and ΔLF/HF ratio. Following the intervention, CVRR, HF, LF/HF ratio, systolic blood pressure (SBP), HR, ΔLF/HF ratio, ΔSBP, and ΔHR improved significantly in the ASH group only.

**Conclusions:**

Four-week supplementation of ASH improved CAF in community-dwelling elderly individuals during resting and standing tests.

**Clinical Trial Registration:**

https://center6.umin.ac.jp/cgi-open-bin/ctr/ctr_view.cgi?recptno=R000031218, UMIN Clinical Trials Registry (UMIN000027251).

## 1 Introduction

The heart rate (HR) is not a constant parameter, and fluctuations occur every beat. Evaluating the beat-to-beat variation in the HR, a parameter referred to as HR variability (HRV), is a reliable and non-invasive approach for assessing cardiac autonomic function (CAF) (1–3). CAF decreases with age (4, 5). Low CAF values are associated with morbidity and mortality following myocardial infarction (6), sudden death (7), and heart disease severity (8). In a 10-year cohort study conducted among community-dwelling elderly individuals, Mäkikallio et al. reported that CAF was an independent predictor of sudden cardiac death (9). Therefore, CAF should be maintained within a normal state in elderly individuals.

Decreased CAF is related to the onset of dizziness (10). Owing to gravitational force, immediately after assuming a standing position, 300 ml–800 ml of blood pools in the skeletal muscles of the lower body. As a consequence, the venous return of blood to the heart decreases, and this results in a diminished stroke volume and blood pressure. In response to this, cardiac sympathetic nervous system (SNS) activity increases, while cardiac parasympathetic nervous system (PNS) activity decreases. These autonomic adjustments increase the HR and cardiac contractility and restore blood pressure to a lower level. However, in elderly individuals with low CAF, this compensation mechanism is not effective during standing. As a result, dizziness is a common finding in elderly individuals (11). A previous epidemiologic survey revealed that approximately 20% of elderly persons aged 65 years and older have experienced dizziness while standing up (12). Ooi et al. also reported that more than 50% of the elderly residents of nursing homes have experienced dizziness (13). Thus, apparent structural disorder in the occurrence of dizziness is a common finding among elderly persons. The occurrence of dizziness while standing is a predictor of vascular death (14) and increases the risk of falls among elderly individuals (15). A clinical practice guideline established by the European Society of Cardiology has also stated that the symptoms of orthostatic hypotension (OH), such as dizziness, are related to unconsciousness (16). Melillo et al. suggested that CAF levels that can increase the risk of falls could reliably be detected (17), and another study reported similar results (18). To prevent falls, a stable CAF level should be maintained in elderly individuals.

*Acanthopanax senticosus* Harms (ASH) is a plant belonging to the Araliaceae family that grows abundantly in various regions of Russia, China, Korea, Southeast Asia, and North Japan (19). The ASH root bark has traditionally been used for nutritional fortification, and ASH supplementation has an anti-stress effect during cold-water immersion restraint in rats (20). In addition, Hartz et al. demonstrated that in patients who experienced chronic fatigue for 6 months, chronic fatigue significantly improved after 2 months of ASH supplementation (21). These results are supported by several previous studies that investigated the physiological effects of ASH in rats and humans (22–24). In other findings, during the novelty-suppressed feeding test, ASH treatment for 1 week signiﬁcantly decreased latency to eat and improved SNS and PNS activity (25). This finding suggests that the anxiolytic effects of ASH result from the regulation of autonomic function. Nevertheless, no previous studies have evaluated the effects of ASH supplementation on CAF in humans. Therefore, this study aimed to examine the effects of continuous ASH supplementation on CAF during tests conducted while resting and standing among the community-dwelling elderly population.

## 2 Methods

### 2.1 Participants

This study was conducted in accordance with the Declaration of Helsinki, and ethical approval was obtained from the Research Ethics Review Committee on Human Subjects of Doshisha University (authorization number: 16008). The trial has been registered in the UMIN Clinical Trials Registry (UMIN000027251). Written consent was obtained from all participants. In addition, participants had 1 month to decide on their willingness to participate.

The sample size was based on a previous study design that measured CAF in elderly individuals by postural changes at rest, tilt-up, and tilt-down, as in the present study (26). Therefore, before conducting the experiments, the appropriate sample size was estimated by power analysis using software G∗power (Version 3.1.9.4) for two-way repeated measures analysis of variance. The effect size f was set to 0.4, alpha error probability to 0.05, beta error probability to 0.95, number of groups to 2, and number of measurements to 2. The calculated total sample size was 24; therefore, 30 subjects were recruited with an anticipation of a 20% dropout.

**Figure 1:**
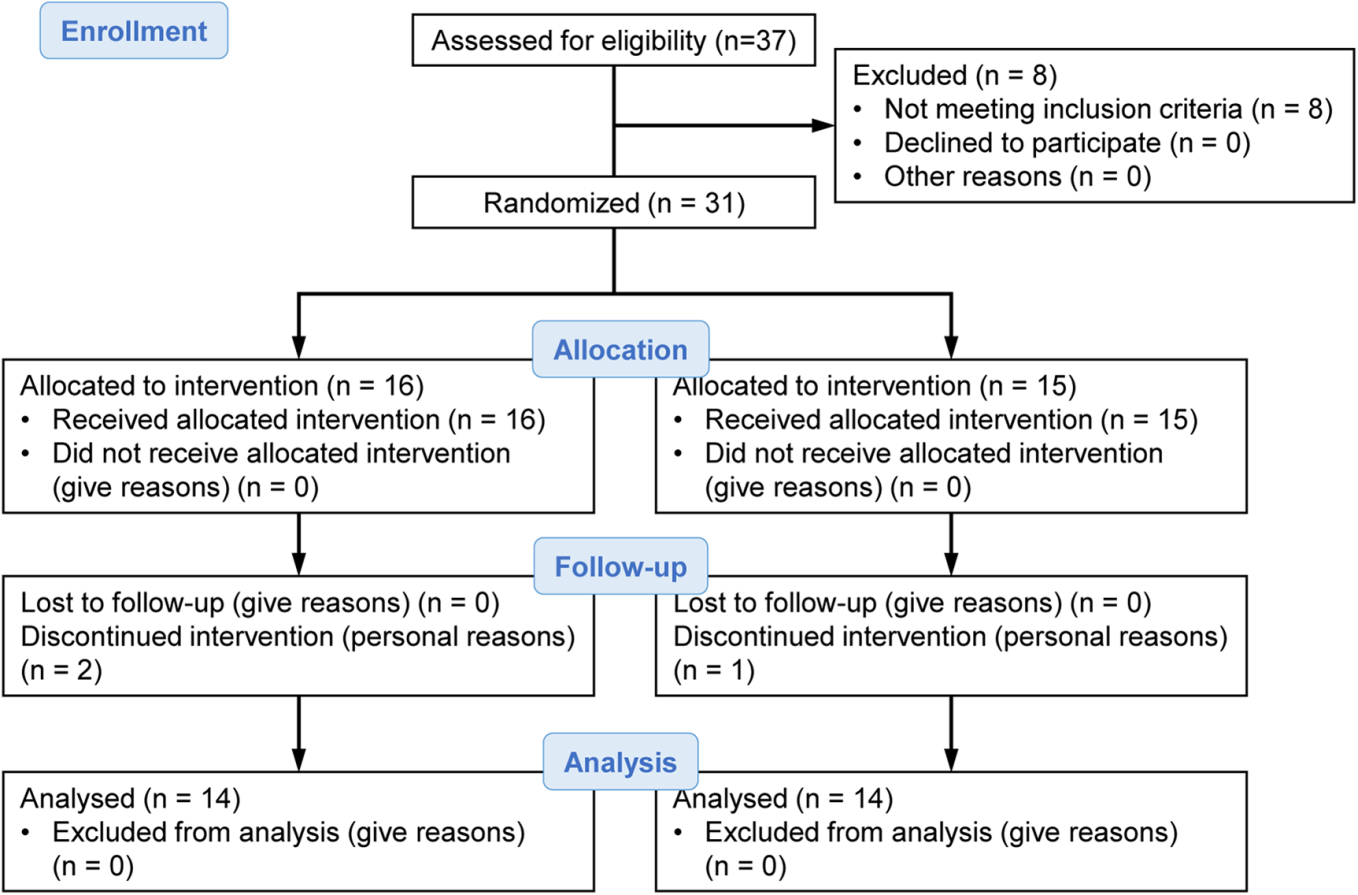
Research flowchart.

Our laboratory recruited participants by posting notifications on the circular community bulletin board and in the flyer of the local neighborhood association of Kyoto city from the end of May to early June 2017. We had 37 applicants, but this study included 31 community-dwelling elderly individuals aged 65–90 years without hypertension, heart disease, or diabetes. Participants were randomly assigned to two groups of 16 and 15 individuals. During the intervention period, two participants from the group of 16 and one participant from the group of 15 interrupted the intervention for personal reasons. Finally, statistical analysis was performed on the two groups of 14 participants each (Figure 1).

### 2.2 Intervention methods

In this double-blind, randomized controlled trial, the participants were assigned to either the ASH group [patients receiving ASH supplements (Sun Chlorella Corp., Kyoto, Japan)] or the placebo group (patients receiving placebo supplements). The randomization code was set using computer-generated random numbers by a researcher who was not engaged in running the trial. The group allocation was blinded for both investigators and participants. Each participant was instructed to consume 12 tablets twice daily (after breakfast and dinner) for 4 weeks between the end of June and the end of July 2017. The compliance rate for all participants was 100%.

Plant powder tablets used in this study were prepared from powdered roots of ASH from Heilongjiang, China. The herb specimen was authenticated by the validation test method of the United States Pharmacopeia that assesses the content of eleutheroside B and E. The tablet (Lot No. 024) was provided by Sun Chlorella Co., Ltd. (Kyoto, Japan). We measured each major component in the tablet using HPLC and an ODS column. These tablets primarily comprised isofraxidin (8.3 mg/100 g), eleutheroside B (74.3 mg/100 g), eleutheroside E (58.2 mg/100 g), eleutheroside B1 (8.5 mg/100 g), and chlorogenic acid (318 mg/100 g).

The participants were provided with a pedometer (HJ-720IT, Omron Health Care Corp., Kyoto, Japan) to monitor their average daily step count during the intervention period. Table 1 presents the ingredients of the ASH and placebo supplements, respectively.

**Table 1:** Ingredients of ASH and placebo.

Ingredients	Ratio of ingredients (%)	Content (mg/table)
ASH		
ASH bulk powder	67.6	135.7
Pullulan	10.1	20.3
Crystalline cellulose	14.9	30.0
Powdered oil	5.0	10.0
Soybean polysaccharides	2.1	4.2
Glycerin	0.3	0.7
Total	100.0	200.9
placebo		
Lactose	91.3	217.4
Caramel	0.5	1.2
Soybean polysaccharides	0.3	0.7
Glycerin	0.1	0.1
Ethanol	0.4	1.0
Purified water	7.5	17.7
Total	100.0	238.0

ASH, *Acanthopanax senticosus* Harms.

### 2.3 Measurement methods

**Figure 2:**
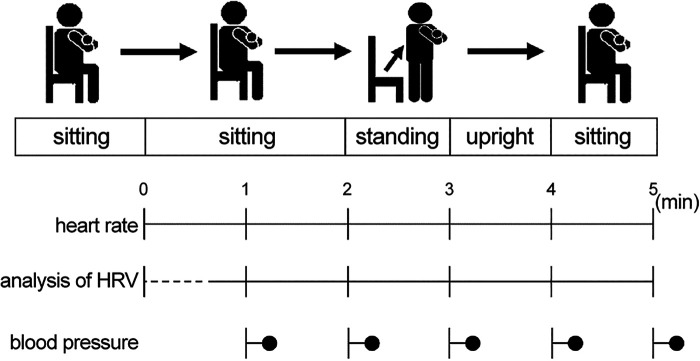
Measurement methods. The participants assumed a seated position for 2 min. Subsequently, they were asked to stand up and remain in a standing position for 2 min, followed by a seated position for 1 min. HRV, heart rate variability.

To measure CAF, an HRV analysis was performed. An autonomic reflex orthostatic tolerance recorder (Kiritsu-Meijin, Crosswell Co., Inc., Yokohama, Japan) was used before and after the 4-week intervention period. This device included an HR monitor (LRR-03, Arm Electronics Corp., Tokyo, Japan) and a blood pressure monitor (TM2584, A&D Company., Ltd., Tokyo, Japan), and recorded the HRV during the R-R intervals on a 2-lead electrocardiogram. The participants were prohibited from performing intense exercise and consuming shellfish, energy drinks, health foods, garlic, and alcohol the day before the measurement. They were instructed to sleep well the day before the measurement, eat breakfast 2 h prior to the measurement, and avoid running while commuting to the laboratory. All measurements were performed in the morning in a laboratory in the Kambaikan at Doshisha University (Karasuma-higashi-iru, Imadegawa-dori, Kamigyo-ku, Kyoto 602-8580, Japan). The participants were allowed to rest sufficiently in a seated position prior to the measurement. During the measurement, the participants were placed in a seated position for 2 min. Subsequently, they were asked to stand up and remain in a standing position for 2 min, followed by a seated position for 1 min (Figure 2). During the measurement, with the aid of an electronic metronome, the breathing of the participants was maintained at 15 breaths/min to reduce the influence of breathing on the results. Using the obtained data, a time domain analysis (27–30) and power spectrum analysis (31, 32) were conducted following the MemCalc method, a new technique for time series analyses.

### 2.4 Measurement of baseline characteristics and HRV

The participants' age, sex, height, weight, and body mass index were obtained. The binary sex categorization (male/female) in this study was determined based on physical and physiological characteristics. Furthermore, we measured the coefficient of variation of the R-R intervals (CVRR), low-frequency (LF: 0.04–0.15 Hz) rate, high-frequency (HF: 0.15–0.40 Hz) rate, LF/HF ratio, systolic blood pressure (SBP) level, diastolic blood pressure (DBP) level, mean blood pressure (MBP) level, and HR. The mean values of these measurement items were assessed while the participants were in a seated position for 2 min. The LF was used as an index of PNS activity and SNS activity. The HF was used as an index of PNS activity. The LF/HF ratio was used as an index of SNS activity (33–35). The changes in each parameter were evaluated before and after standing. The ΔCVRR, ΔLF, ΔHF, ΔLF/HF ratio, ΔSBP, ΔDBP, ΔMBP, and ΔHR were defined as the difference between the mean values obtained in a standing position for 1 min and those obtained in a seated position for 2 min (e.g., ΔCVRR = CVRR in a standing position for 1 min—CVRR in a seated position for 2 min).

### 2.5 Statistics

The values are expressed as mean ± SD. The statistical differences between the groups were assessed using Student's unpaired *t*-test and a *χ*^2^-test before the intervention. A two-way analysis of variance with repeated measures was used to assess the interaction effects on the measured parameters between the ASH and placebo groups. Parameters for which the ANOVA results were significant were compared to baseline values for the ASH and placebo groups using a paired Student's *t*-test. All statistical analyses were performed using SPSS statistics 25 (IBM, Tokyo, Japan). A *p*-value of <0.05 was considered signiﬁcant.

## 3 Results

### 3.1 Baseline characteristics and daily step counts during the intervention period

During the intervention period, three participants withdrew from the study for personal reasons, leaving a total of 28 participants [mean ± standard deviation (SD) = 72.5 ± 4.5 years] for analysis. No significant differences were observed in male-to-female ratio and any of the measurement items between the ASH and placebo groups before the intervention (Tables 2, 3). During the intervention period, no significant differences were observed in the daily step counts between the ASH (5,985 ± 2,167 steps/day) and placebo (6,803 ± 2,833 steps/day) groups.

**Table 2:** Comparison of characteristics between the ASH and placebo groups.

	ASH group	Placebo group	*t*	*p*
	*n* = 14 (M/F: 3/11)	*n* = 14 (M/F: 5/9)		
Age (years)	72.8 ± 4.8	72.1 ± 4.2	0.42	0.682
Height (cm)	153.4 ± 6.6	156.6 ± 10.4	−0.97	0.340
Weight (kg)	57.6 ± 9.7	53.1 ± 9.6	1.20	0.241
BMI (kg/m^2^)	24.3 ± 2.9	21.7 ± 3.6	1.41	0.060

Data are expressed as mean ± standard deviation. ASH, *Acanthopanax senticosus* Harms; BMI, body mass index.

**Table 3:** Group-time interactions on heart rate variability between the ASH group and placebo group.

	ASH group	Placebo group	Group × time	
	(*n* = 14)	(*n* = 14)	*F*	*p*
CVRR (%)				
Before	2.2 ± 1.2	2.4 ± 1.1	8.16	0.008
After	2.8* ± 1.8	2.0 ± 0.7		
LF (ms^2^)				
Before	143.6 ± 140.1	132.0 ± 52.3	4.07	0.054
After	151.8 ± 132.5	89.4 ± 64.5		
HF (ms^2^)				
Before	75.6 ± 65.4	101.5 ± 84.5	12.0	0.002
After	174.5** ± 120.1	81.3 ± 71.9		
LF/HF ratio				
Before	1.9 ± 1.6	1.3 ± 1.0	2.86	0.103
After	0.9 ± 0.7	1.1 ± 0.8		
HR (bpm)				
Before	72.6 ± 10.3	73.7 ± 7.7	0.02	0.878
After	67.6 ± 6.8	69.2 ± 9.0		
SBP (mmHg)				
Before	126.0 ± 15.0	122.3 ± 13.0	0.10	0.758
After	121.0 ± 13.2	118.6 ± 12.8		
DBP (mmHg)				
Before	75.6 ± 9.4	76.6 ± 6.4	0.01	0.928
After	73.1 ± 8.1	74.4 ± 8.4		
MAP (mmHg)				
Before	92.4 ± 10.9	91.9 ± 8.1	0.05	0.823
After	89.1 ± 9.5	89.1 ± 9.2		
ΔCVRR (%)				
Before	1.8 ± 1.1	1.3 ± 1.1	1.24	0.276
After	1.4 ± 0.9	1.3 ± 0.8		
ΔLF (ms^2^)				
Before	27.8 ± 36.7	103.9 ± 94.8	1.02	0.321
After	104.7 ± 84.9	107.9 ± 99.3		
ΔHF (ms^2^)				
Before	−18.2 ± 101.5	15.9 ± 40.1	2.79	0.107
After	−66.9 ± 145.7	19.7 ± 69.4		
ΔLF/HF ratio				
Before	1.5 ± 1.4	1.2 ± 0.8	8.34	0.008
After	2.9** ± 1.2	1.0 ± 0.7		
ΔHR (bpm)				
Before	5.0 ± 3.3	4.5 ± 3.4	1.23	0.278
After	6.4 ± 3.5	4.7 ± 3.1		
ΔSBP (mmHg)				
Before	−4.6 ± 3.7	−4.4 ± 2.1	1.93	0.177
After	3.6 ± 3.6	−3.6 ± 2.6		
ΔDBP (mmHg)				
Before	0.02 ± 4.4	−2.7 ± 5.7	0.02	0.903
After	0.14 ± 4.7	−2.1 ± 8.3		
ΔMAP (mmHg)				
Before	−1.5 ± 4.3	−3.3 ± 7.1	0.05	0.818
After	1.1 ± 5.4	−2.6 ± 8.9		

Data are expressed as mean ± standard deviation. ASH, *Acanthopanax senticosus* Harms; CVRR, coefficient of variation of R-R intervals; DBP, diastolic blood pressure; HF, high frequency (0.15–0.40 Hz); HR, heart rate; LF, low frequency (0.04–0.15 Hz); MBP, mean blood pressure; SBP, systolic blood pressure. All the parameters (ΔCVRR, ΔLF, ΔHF, ΔLF/HF ratio, ΔSBP, ΔDBP, ΔMBP, and ΔHR) were defined as the difference between the mean values obtained in a standing position for 1 min and in a seated position for 2 min.

* *p* < 0.05.

** *p* < 0.01, vs. ASH group before the intervention.

### 3.2 Changes in measurement items before and after the intervention

The two-way analysis of variance revealed significant group-time interactions in the CVRR, HF, and ΔLF/HF ratio (Table 3). Following the intervention, the CVRR, HF, and ΔLF/HF ratio significantly improved in the ASH group (Table 3). Similar changes were not observed in the placebo group.

## 4 Discussion

In this study, significant group-time interactions were observed in the CVRR, HF, and ΔLF/HF ratio Following the intervention period, in the ASH group, the CVRR, HF, and ΔLF/HF ratio significantly increased. In several previous studies, CAF decreased with age (36, 37). However, low CAF results not only from aging but also various diseases; patients with obesity and diabetes have a low CAF (38–40). In the Framingham Heart Study, low HF was associated with all-cause mortality in elderly individuals, even after adjusting the prevalence for age, sex, smoking history, diabetes, and heart disease (41). Therefore, maintaining normal CAF states among elderly individuals is important, as CAF reduction is associated with various pathologies and mortality. Notably, the CVRR, HF, and ΔLF/HF ratio were significantly increased in the ASH group after the intervention period. Therefore, continuous ASH supplementation may potentially improve CAF in a resting state among elderly individuals. The HR is controlled by PNS and SNS activities. In a resting state, PNS activity increases, while SNS activity decreases. In an active state, SNS activity increases, while PNS activity decreases. The PNS and SNS enable HR to fluctuate appropriately during various human activities (42, 43). In this study, the HF increased in the ASH group following the intervention period. Considering that the HF was used as an index of PNS activity, this suggests improvement in resting state HR.

Furthermore, CAF is associated with blood pressure regulation (44). In a previous study, reduced CAF was associated with the incidence of hypertension in men during a follow-up period of 4 years, even after adjusting for factors associated with hypertension (45). Additionally, individuals with high SBP and DBP levels exhibit decreased PNS activity and increased SNS activity during the resting state (46, 47). Based on the above findings, continuous ASH supplementation can properly regulate not only the CAF level but also blood pressure levels in elderly individuals.

When an adult assumes a standing position, 300 ml–800 ml of blood pools in the lower extremities, and the venous return of blood to the heart and blood pressure decrease (48, 49). In this situation, the SNS and PNS decrease the blood pressure level (33). In contrast, CAF may not be effective when elderly individuals and patients with diabetes assume a standing position, and blood pressure may not return to normal (4). Therefore, the incidence of dizziness is relatively high among elderly individuals and patients with diabetes. Kawaguchi et al. clarified that, when used as an index of SNS activity, the LF/HF ratio increased significantly in young participants immediately after assuming a standing position, while it only increased in elderly participants following a delay after assuming a standing position (50). Our results revealed significant group-time interactions in the CVRR, HF, and ΔLF/HF ratio. A decrease in PNS activity and an increase in SNS activity are related to an elevation in HR and blood pressure levels (10, 51). Therefore, continuous ASH supplementation increased the CAF and blood pressure responsiveness among elderly participants while they were in a standing position.

In the ASH group, an increase in the HF while in a resting state significantly improved the ΔLF/HF ratio. Taylor et al. clarified that HF decreases immediately after a healthy adult performs an exercise, but the decrease is mild in elderly individuals. A significant positive correlation was previously observed between the HF at rest and the ΔHR and ΔLF/HF ratio from a resting to an active state (52). Accordingly, the decrease in the HF at rest contributes to the decrease in the reactivity of the HR and SNS activity while standing. Based on the above findings, the increase in the HF at rest in the ASH group contributed to the improvement in the ΔLF/HF ratio reported in this study. Additionally, a previous study examining the effects of hot spring foot bathing on CAF among frail, elderly Japanese individuals using the same standing test as in this study found no improvement in CAF. Therefore, we suggest that ASH intake among elderly individuals is an effective means of improving CAF (53).

In previous studies, treatment with ASH reduced the cardiovascular responses to stress in healthy young individuals (24), and ASH treatment for 1 week signiﬁcantly increased the HF in rats during an improved elevated beam-walking test (25). However, only a few studies have investigated the effects of continuous ASH supplementation on CAF and no study has analyzed the effects of ASH supplementation on CAF in humans. Our results suggest that continuous ASH supplementation may improve CAF during resting and standing states in elderly individuals.

### 4.1 Limitations and strengths of the trial

Certain limitations must be taken into account for the interpretation and generalization of this study's results, such as the relatively small sample size and brief study duration. Therefore, future research should be conducted to recruit a larger sample of participants. In addition, longer duration trials of adequate sample size with a clinically significant margin should be designed to determine the equivalence of the interventions. Secondly, the mechanism behind the improvement in CAF was not clearly explained by our results, similar to previous studies. However, ASH plays a role in the body's coping mechanism during stress via a brain noradrenergic mechanism. Notably, the main components of ASH, which include syringin, chlorogenic acid, eleutheroside E, and isofraxidin, were involved in that action (54–56).

Further, the sudden drop in blood pressure levels when assuming a standing position is not necessarily caused by a decrease in CAF, and several factors, such as the progression of atherosclerosis and reduced baroreceptor sensitivity, have an effect (10, 49, 57, 58). Recent evidence indicates that ASH has the potential to improve blood pressure (BP) and arterial stiffness via endothelial eNOS activation in healthy adults who smoke and have a tendency toward elevated BP or blood lipid parameters (59). Therefore, the improvement in blood pressure responsiveness during the standing test in our study may not have been due to an improvement in CAF. However, CAF reduction and the occurrence of dizziness are associated with various pathologies and mortality, and CAF should be maintained at a normal state in elderly individuals. The results of this study have elucidated the effects of ASH on CAF during resting and standing states among elderly individuals and have demonstrated the potential clinical applicability of ASH.

In conclusion, this study examined the effects of continuous ASH supplementation on CAF during resting and standing tests in community-dwelling elderly individuals. The results of this study have demonstrated that supplementation with ASH over a 4-week period improved the CAF and blood pressure responsiveness in community-dwelling elderly individuals during resting and standing tests.

## Data Availability

The raw data supporting the conclusions of this article will be made available by the authors, without undue reservation.
